# Sciatic nerve course in adult patients with unilateral developmental dysplasia of the hip: implications for hip surgery

**DOI:** 10.1186/1471-2482-15-14

**Published:** 2015-01-31

**Authors:** Ruiyu Liu, Jiawei Liang, Kunzheng Wang, Xiaoqian Dang, Chuanyi Bai

**Affiliations:** Department of Orthopaedic, the Second Hospital affilicated to medical college Xi’an Jiaotong University, Xi’an, Shaanxi 710004 P. R. China

**Keywords:** Sciatic nerve course, Injury, Developmental dysplasia of the hip, Hip surgery, Posterolateral approach

## Abstract

**Background:**

Sciatic nerve injury is a disastrous adverse complication of surgery and can cause debilitating pain, functional impairment and poor quality of life. Patients with developmental dysplasia of the hip (DDH) have a high incidence of sciatic nerve injury after total hip arthroplasty (THA). A better understanding of the course of the sciatic nerve in patients with DDH may help minimise the risk of sciatic nerve injury after THA.

**Methods:**

A total of 35 adult patients with unilateral DDH were enrolled in this retrospective study. We reviewed the patients’ computed tomography (CT) scans, which included the area from the iliac crest to below the lesser trochanter. The distance between the sciatic nerve and regional anatomic landmarks in four different sections on CT scans was measured to identify the course of the sciatic nerve.

**Results:**

The distance from the sciatic nerve to the spine’s midline was shorter on the affected side than on the healthy side (p < 0.05); the same difference was also detected in the distance to the ilium/ischium outside the true pelvis (p < 0.05). The distance to the greater trochanter was longer on the affected side (p < 0.05). However, the two sides showed no significant difference in the distance from the sciatic nerve to the lesser trochanter (p > 0.05).

**Conclusions:**

For patients with unilateral DDH, the sciatic nerve was located near the ischium and ilium but relatively far from the femur of the affected hip joint, compared to its location on the healthy side. These findings reveal that sciatic nerve becomes shorter in the affected low-limb and is relatively unlikely to be directly injuried using the posterolateral approach in patients with unilateral DDH.

## Background

Surgery-related nerve injury causes debilitating pain and functional impairment in patients. It severely affects a patient’s postoperative recovery and reduces health-related quality of life [1, 2]. Sciatic nerve injury accounts for more than 80% of nerve injuries in total hip arthroplasty (THA), with an incidence of approximately 1% [3]. The incidence of sciatic nerve injury reaches up to 5.2% in THA performed for developmental dysplasia of the hip (DDH) [4]. Limb lengthening is believed to be one of the major causes of sciatic nerve injury [5–8]. However, Eggli [9] and other researchers [10, 11] have found that the incidence of nerve damage is not statistically correlated with the amount of limb lengthening, but rather with the difficulty of the surgery.

The course of the sciatic nerve through the region of the healthy hip has been widely investigated [12, 13], and valuable information has been obtained regarding the protection of the sciatic nerve during surgery on normally developed hip joints. Nevertheless, the sciatic nerve may deviate from its normal course due to femoral subluxation, pelvic tilt, and/or muscle contracture in patients with DDH. Thus, THA in adult patients with DDH is a technically challenging procedure and is associated with a high risk of sciatic nerve injury during surgery. A clear understanding of the local anatomy of the sciatic nerve and careful surgical technique can help to reduce the risk of sciatic nerve injury. Unfortunately, little information is available on the course of the sciatic nerve in patients with DDH.

Growing evidence indicates that injuries to the sciatic nerve occur in the hip region [5, 11, 14]. In this study, we investigated the course of the sciatic nerve in the hip region in adult patients with DDH. We have investigated the acetabular bone stock [15] and changes in the gluteus medius muscle in patients with unilateral DDH [16]. We retrospectively analysed the computed tomography (CT) scans of patients with unilateral DDH to obtain information about the course of the sciatic nerve with the goal of improving THA outcomes and avoiding sciatic nerve injuries.

## Methods

### Patients

Thirty-five patients with unilateral DDH were retrospectively analysed in this study. The patients included 6 men and 29 women with an average age of 49 years (range 36–63 years), a mean weight of 56 kg (range 50–72 kg), and a mean height of 159 cm (range 156–173 cm). None of the patients underwent osteotomy for treatment or hip correction. We reviewed patient CT scans that had been collected between 2007 and 2012 for morphological evaluation of the acetabular and proximal femur before total hip arthroplasty. Routine radiographs of the anteroposterior pelvis were also collected for diagnosis and classification of the disease. DDH has been defined by a centre edge (CE) angle < 20 degrees on radiographs [17]. According to the Crowe classification for adult DDH [18], 19 patients were in DDH class II, and 16 patients were in class III. Written consent was obtained from each patient, and the study was approved by the Ethical Committee of the Second Affiliated Hospital of Xi’an Jiaotong University, Xi’an, China.

### Computed tomography scans

Patients were scanned with a Phillips MX 8000 spiral CT scanner (Phillips Medical Systems, Cleveland, Ohio, USA) in the supine position, with the pelvis in the neutral position, the lower limb placed in internal rotation, and the patella facing forward. CT scan parameters were as follows: slice thickness 2 mm; slice interval 2 mm; voltage 120 kV; current 100 mA;and time 0.5 s. Transverse images of the area from the iliac crest to below the lesser trochanter were obtained for each patient. The original images were transmitted in Digital Imaging and Communications in Medicine (DICOM) format (512 × 512 pixels) and analysed using a CT workstation with a window and level optimised for soft tissue display. The sciatic nerve was identified based on its shape of the cross-section and its adjacent anatomical relationships in the same plane, further identification of it was done by combining several images of adjacent planes.

### Localization of the sciatic nerve

**Figure 1:**
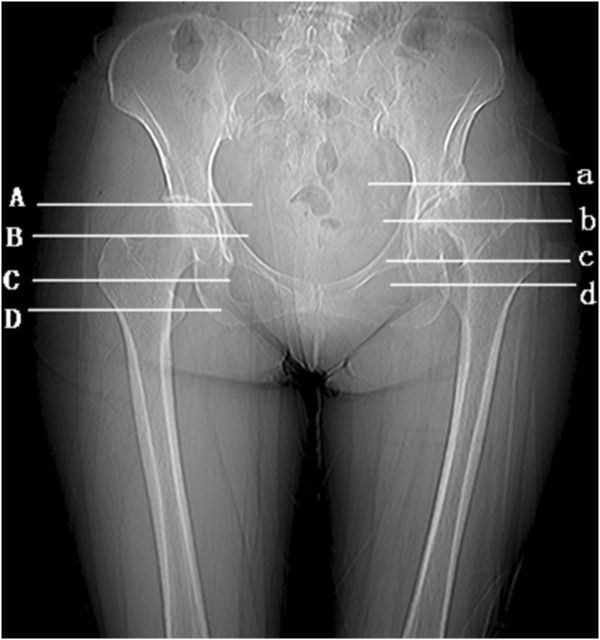
**Diagram of the measurement planes on pelvic radiographs.**

The distances from the sciatic nerve to the regional anatomic landmarks on each plane were measured to locate the path of the sciatic nerve as it passes through the gluteal region of the DDH patients. The transverse plane which connects the two iliac crests serves as the reference plane, and then four transverse planes that run in parallel to the reference plane were selected for measurement. The four transverse planes crossed the superior border of the femoral head (Plane A), the superior border of the greater trochanter (Plane B), the inferior border of the greater trochanter (Plane C) and the centre of the lesser trochanter (Plane D) (shown in Figure 1).

#### In Plane A

The sciatic nerve was located in the pelvis, directly behind the lower edge of the piriformis. We measured the linear, horizontal, and perpendicular distances between the sciatic nerve and the trailing edge of the ilium.

#### In Plane B

The sciatic nerve ran between the gemellus superior muscle and the gluteus maximus muscle. The following linear, horizontal, and perpendicular distances were measured: from the sciatic nerve to the trailing edge of the ilium and from the sciatic nerve to the greater trochanter.

#### In Plane C

The gemellus inferior muscle was situated between the greater trochanter and the ischial tuberosity. The sciatic nerve was between the gemellus inferior muscle and the gluteus maximus muscle. We measured the linear, horizontal, and perpendicular distances from the sciatic nerve to the trailing edge of the ischium, and from the sciatic nerve to the trailing edge of the greater trochanter.

#### In Plane D

The sciatic nerve was between the quadratus femoris muscle and the gluteus maximus. The following linear, horizontal, and perpendicular distances were measured: from the sciatic nerve to the trailing edge of the ischium and from the sciatic nerve to the lesser trochanter.

The distances between the sciatic nerve and the pelvic midline in the four transverse planes were also measured.

In order to keep the consistency in the referenced bony landmark, measurements of distance between the sciatic nerve and the pelvic midline, and the bone landmark of pelvis on the affected side were performed at the same levels as those of Planes A to D on the healthy side. The distance between the sciatic nerve and the landmark of the femur on the affected and healthy side were measured on the planes referenced to their respective landmarks of the femur, that is, Planes b-d on the affected side and Planes B to D on the healthy side (shown in Figure 1).

**Figure 2:**
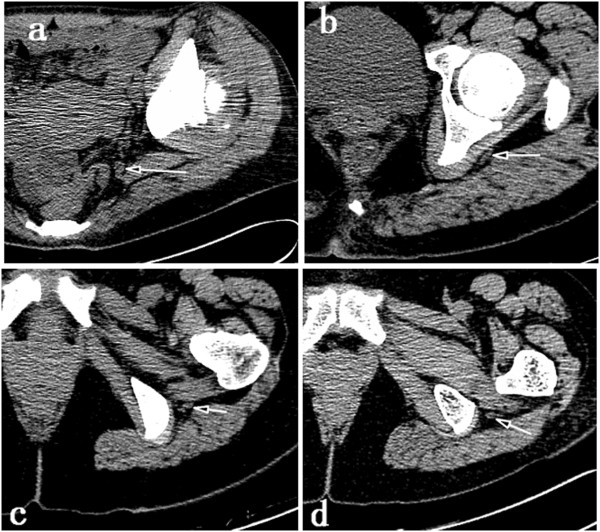
**Axial CT scans demonstrating the planes used for evaluation of the sciatic nerve (indicated by arrow) in a cranio-caudal direction.** The sciatic nerve was evaluated in planes perpendicular to the spine and crossing the superior border of the femoral head **(a)**, the superior border of the greater trochanter **(b)**, the inferior border of the greater trochanter **(c)**, and the centre of the lesser trochanter **(d)**.

**Figure 3:**
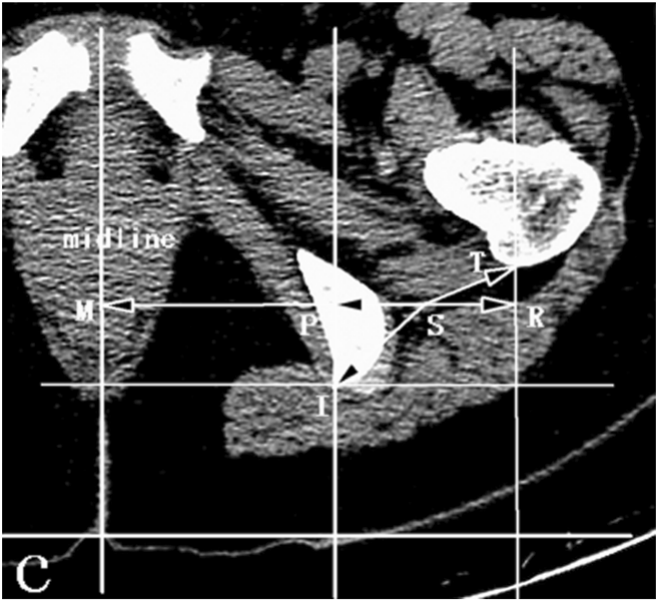
**Schematic measurement of the sciatic nerve’s orientation in the horizontal plane crossing the top of the greater trochanter.** SM horizontal distance between the sciatic nerve and pelvic midlineSI linear distance between the sciatic nerve and the trailing edge of the ischium SP horizontal distance between the sciatic nerve and the trailing edge of the ischium PI perpendicular distance between the sciatic nerve and the trailing edge of the ischium ST linear distance between the sciatic nerve and the trailing edge of the greater trochanter SR horizontal distance between the sciatic nerve and the trailing edge of the greater trochanter RT perpendicular distance between the sciatic nerve and the trailing edge of the greater trochanter.

These measurements are shown in Figure 2 and Figure 3.

The distances were measured using the calibrated measuring tool of the computer workstation (Phillips MX LiteView1.0, Phillips/Marconi Marconi Medical Systems Inc., Cleveland, Ohio, USA). All measurements were performed independently by two experienced musculoskeletal radiologists who were blinded to their own data and to each other’s data. The first investigator performed the measurements twice at an interval of three weeks, and the second one performed the measurements only once. Intra-observer and inter-observer variations in distance measurements were assessed using intra-class correlation coefficients.

### Statistics

All data analyses were performed using SPSS 13.0 (SPSS, Chicago, IL, USA). Comparisons between the affected hip and the healthy side were performed using the Wilcoxon signed rank test. Statistical significance was set at p < 0.05.

## Results

All patients in the present study underwent total hip arthroplasty. One patient experienced sciatic nerve palsy after THA but recovered six months after surgery.

The intra-observer and inter-observer repeatability of measurements was acceptable. The intra-observer correlation coefficients for measurements ranged from 0.89 to 0.93 and the inter-observer correlation coefficients ranged from 0.81 to 0.83.

There was no statistically significant difference between the affected and healthy hips of the patients in terms of the horizontal distance from the sciatic nerve to the spinal midline in Plane A. However, this distance on the affected side was significantly shorter than on the healthy side in Planes B, C, and D (shown in Table 1).

**Table 1:** **Distance between the sciatic nerve and the pelvic midline in DDH patients**

	Affected side (mm)	Healthy side (mm)	Difference (%)	P-value
Plane A	47.7(5.3)	50.7(8.9)	−5.9	0.123
Plane B	65.7(12.3)	76.3(9.2)	−15.8	0.006
Plane C	84.7(8.6)	94.4(7.8)	−12.3	0.010
Plane D	92.3(6.3)	96.8(7.8)	−4.61	0.029

(−) indicates a reduction in distance on the affected side.

Significant differences were observed in the linear and horizontal distances from the sciatic nerve to the trailing edge of the ilium/ischium between the affected and healthy hips in the four planes (p < 0.05). In Plane A, the linear and horizontal distances on the affected side increased by 46.5% and 68.6%, respectively, when compared to those on the healthy side. However, the linear and horizontal distances on the affected side decreased, respectively, by 28.7% and 38.3% in Plane B, by 25.5% and 24.2% in Plane C, and by 19.3% and 23.1% in Plane D (shown in Table 2).

**Table 2:** **Distance between the sciatic nerve and the ilium/ischium in DDH patients**

	Affected side (mm)	Healthy side (mm)	Difference (%)	P-value
**Plane A**				
linear	24.1(7.3)	16.4(6.9)	46.5	0.001
horizontal	19.3(8.1)	11.5(6.5)	68.6	0.001
perpendicular	11.6(6.3)	10.4(4.3)	12.1	0.283
**Plane B**				
linear	16.2(6.7)	23.0(7.0)	−28.7	0.042
horizontal	12.7(8.2)	20.6(7.6)	−38.3	0.013
perpendicular	7.3(4.9)	5.6(4.8)	26.3	0.274
**Plane C**				
linear	25.5(6.8)	34.3(10.9)	−25.5	0.011
horizontal	21.2(5.6)	28.0(8.3)	−24.2	0.030
perpendicular	11.2(7.5)	16.4(8.9)	−33.1	0.028
**Plane D**				
linear	26.7(9.0)	33.1(5.5)	−19.3	0.014
horizontal	21.8(7.4)	28.4(6.6)	−23.1	0.018
perpendicular	12.3(7.1)	13.4(6.8)	−8.1	0.593

(−) indicates a reduction in distance on the affected side.

The linear and horizontal distances from the sciatic nerve to the greater trochanter were significantly larger on the affected side than on the control side in Planes B and C (p < 0.05). The linear and horizontal distances on the affected side increased by 18.95% and 23.91% in Plane B and by 47.60% and 51.36% in Plane C, respectively. Nonetheless, the linear and horizontal distances to the lesser trochanter on the affected side decreased by 2.21% and 13.80% in Plane D, but the differences between the two sides were not significantly different (p > 0.05) (as shown in Table 3).

**Table 3:** **Distance between the sciatic nerve and the femur in DDH patients**

	Affected side (mm)	Healthy side (mm)	Difference (%)	P-value
Distance to the superior border of the greater trochanter				
**Plane B**				
linear	58.9(19.1)	49.54(17.1)	18.95	0.024
horizontal	54.8(19.6)	44.25(16.3)	23.91	0.013
perpendicular	18.1(8.0)	21.22(6.6)	−14.80	0.125
Distance to the inferior border of the greater trochanter				
**Plane C**				
linear	35.4(11.9)	23.9(9.8)	47.60	0.009
horizontal	30.5(13.8)	20.2(10.8)	51.36	0.007
perpendicular	13.8(8.3)	10.2(6.4)	31.13	0.091
Distance to the lesser trochanter				
**Plane D**				
linear	21.2(5.0)	21.2(5.9)	−2.21	0.781
horizontal	8.9 (6.5)	10.4(8.1)	−13.80	0.583
perpendicular	17.7(6.3)	16.2(6.6)	−9.46	0.416

(−) indicates a reduction in distance on the affected side.

When compared to the control side, the affected hip showed an obvious decrease in the horizontal distance from the sciatic nerve to the outer edge of the ischium but a remarkable increase in the horizontal distance from the sciatic nerve to the outer edge of the greater trochanter.

The affected and control hips showed no significant differences in the perpendicular distance from the sciatic nerve to the surrounding bony landmarks in all planes (p > 0.05) (Table 2).

## Discussion

Few reports have been published on the course of the sciatic nerve in patients with DDH, due to the difficulty in obtaining cadaveric donors with DDH. Fortunately, researchers have begun to use in vivo imaging to study the course of the sciatic nerve. Using MRI, Birke et al. [19] measured the distance from the sciatic nerve to the infracotyloid groove in children, and located the position of the sciatic nerve in different hip positions. Their study provided guidance for orthopaedic surgery of the hip and pelvis. Tagliafico et al. [20] and Hurd et al. [21] also used in vivo imaging to identify aetiologic factors for sciatic nerve palsy following total hip arthroplasty. In this study, the position of the sciatic nerve in the hip was identified using CT images of DDH patients, CT images have been used to observe the nerve in the hip region [22, 23].

Anatomic identification of the sciatic nerve was performed based on the anatomic features of the sciatic nerve in the hip region. All measurements were independently obtained by two experienced musculoskeletal radiologists. Therefore, the results of this study are reliable.

The posterolateral approach can provide optimal exposure and is widely used in THA for patients with DDH. However, this technique may lead to a high risk of sciatic nerve injury because the approach to the hips is adjacent to the path of the sciatic nerve. This study found that the distance between the sciatic nerve and the greater trochanter was longer on the affected side than on the healthy side. Therefore, the possibility that the posterolateral approach caused direct sciatic nerve injury was relatively low in patients with unilateral DDH.

Over-tension is thought to be one of the pathological mechanisms underlying sciatic nerve palsy, and excessive tension due to limb lengthening may be one of the main causes of sciatic nerve palsy [5–8]. It has been suggested that the hip should be reconstructed in its anatomical position in order to obtain an optimal biomechanical result in DDH patients. This type of reconstruction is accompanied by limb lengthening. Limb lengthening of more than 4 cm is strongly associated with an increased risk of sciatic nerve palsy during THA [6, 7]. On the affected side, the path of the sciatic nerve is not displaced correspondingly with the proximal and lateral displacement of the femur. Thus, the length of the sciatic nerve decreases on the affected side compared to the healthy side, and the extension of the sciatic nerve is limited when the affected hip is reconstructed. Over-lengthening of the affected lower limb should be avoided in order to reduce the risk of sciatic nerve injury during THA. However, Eggli and other researchers believe that sciatic nerve injury is not related to the amount of lengthening, but rather to the difficulty of surgery [9–11]. THA is technically challenging in DDH patients because the acetabular reconstruction must be reoriented and the limb length discrepancy must be eliminated or greatly reduced. The difficulty of THA increases the incidence of direct or indirect mechanical sciatic nerve injury. Thus, we believe that both over-lengthening of the lower limb and the difficulty of surgery are risk factors for sciatic nerve injury in patients with DDH, attention must be paid to these risk factors to avoid nerve injury.

Acetabular osteotomy is commonly used as a surgical treatment for hip dysplasia in adolescents and young adults. It is a technically demanding procedure for surgeons in that the surgery is performed under fluoroscopy. Published studies have reported the occurrence of sciatic nerve injuries in patients undergoing periacetabular osteotomy [24–26]. Familiarity with the course of the sciatic nerve adjacent to the acetabulum is essential for avoiding nerve injury during hip reconstruction. Our study demonstrated that the distance between the sciatic nerve and the spinal midline was decreased in the hip affected by DDH. Moreover, the sciatic nerve was relatively far away from the ilium before passing through the pelvis but was close to the ilium/ischium after threading through the pelvis. The identification of the course of the sciatic nerve in this study may help to reduce the risk of sciatic nerve injury during hip reconstruction.

## Conclusion

The sciatic nerve was closer to the ilium and ischium but farther from the femur in the hip region in adult patients with unilateral DDH. The changes in the course of the sciatic nerve may be associated with femoral subluxation, which can cause the shortening of the sciatic nerve in patients with unilateral DDH. These findings suggest that the surgical approach was relatively unlikely to cause direct sciatic nerve injury in patients with unilateral DDH. A high incidence of sciatic nerve injury may be related to the over-lengthening of the low-limb in total hip anthroplasty and the complexity and difficulty in performing the surgery.

## Abbreviations

THA: Total hip arthroplasty
DDH: Developmental dysplasia of the hip
CT: Computed tomography
MRI: Magnetic resonance imaging.
